# Observations to Prove That in Cases Where the Upper Extremities Present, at the Time of Birth, the Delivery May Be Effected by the Spontaneous Evolution of the Child

**Published:** 1784

**Authors:** 


### SECTION II.

### Essays and Observations.

I. *Obſervations to prove that in caſes where the upper extremities preſent, at the time of birth, the delivery may be effected by the ſpontaneous evolution of the child. Communicated in a letter to Dr.* Simmons, *F. R. S. by* Thomas Denman, *M. D. Licentiate in Midwifery of the Royal College of Phyſicians, Phyſician-man-midwife to the Middleſex Hoſpital, and Teacher of Midwifery in London.*

IN the preſentation of the ſuperior extremities of children, at the time of birth, it has been an opinion, I believe, univerſally adopted, that women would die undelivered, if they were not relieved by art. Being informed that the following caſes, which are contradictory to this opinion, have been miſrepreſented, and ſometimes miſquoted, I beg the favour of you to publiſh publiſh this ſhort account in the London Medical Journal.

### Case I.

In the year 1772, I was called to a poor woman in Oxford-ſtreet, who had been in labour all the preceding night, under the care of a midwife. Mr. Kingſton, now living in Charlotte-ſtreet, and Mr. Goodwin, ſurgeon, at Wirkſworth, in Derbyſhire, who were at that time ſtudents in midwifery, had been ſent for, ſome hours before I was called. The arm of the child preſenting, they attempted to turn and extract it by the feet, but the pains were ſo ſtrong as to prevent the introduction of the hand into the uterus. I found the arm much ſwelled, and puſhed through the external parts in ſuch a manner, that the ſhoulder nearly reached the perinæum. The woman ſtruggled vehemently with her pains, and during their continuance, I perceived the ſhoulder of the child to deſcend. Concluding that the child was ſmall and would paſs, doubled, through the pelvis, I deſired one of the gentlemen to ſit down to receive it, but the friends of the woman would not permit me to move. I remained by the bed-ſide till the child was expelled,

 and and I was very much furprifed to find, that the breech and inferior extremities were expelled before the head, as if the cafe had originally been a prefentation of the inferior extremities.

The child was dead, but the mother recovered as foon and as well as fhe could have done after the moft natural labour.

### Case II.

In the year 1773, I was called to a woman in Caftle-ftreet, Oxford-market, who was attended by a midwife. Many hours after it was difcovered that the arm of the child prefented. Mr. Buroffe, furgeon, in Poland-ftreet, was fent for, and I was called into confultation. When I examined, I found the fhoulder of the child preffed into the fuperior aperture of the pelvis. The pains were ftrong and returned at fhort intervals. Having agreed upon the neceffity of turning the child and extracting it by the feet, I fat down and made repeated attempts to raife the fhoulder, with all the force which I thought could be fafely ufed; but the action of the uterus was fo powerful that I was obliged to defift. I then called to mind the circumftances of the cafe before related, mentioned them to Mr. Buroffe, and propofed that we fhould wait for the effect, which which a continuance of the pains might produce, or till they were abated, when the child might be turned with lefs difficulty. No further attempts were made to turn the child. Then every pain propelled it lower into the pelvis, and in little more than one hour the child was born, the breech being expelled, as in the firft cafe.

This child was alfo dead, but the mother recovered in the moft favourable manner.

Having been prepared for obferving the progrefs of this labour, I underftood it more clearly, and attempted to explain both in my lecture on the fubject, and in the aphorifms which were printed for the ufe of the ftudents in the fame year, that is, 1773, my opinion of the manner in which the body of the child turned as it were, upon its own axis. I alfo pointed out the circumftances, in which, I fuppofed, the knowledge of the fact might be rendered ufeful in practice; but with great circumfpection.

### CASE III.

January the 2d, 1774, I was called to Mrs. D——, who keeps a toyfhop in Crown-court, Windmill-ftreet. She had been a long time in labour, and the arm of the child prefented.

The late Mr. Euftace had been called on the preceding evening, and had made attempts to turn the child, which he had continued for ſeveral hours without ſucceſs. I was ſent for about one o'clock in the morning, and on examination found the arm puſhed through the external parts, the ſhoulder preſſing firmly upon the perinæum. The exertions of the mother were wonderfully ſtrong. I ſat down while ſhe had two pains, by the latter of which the child was doubled, and the breech expelled. I extracted the ſhoulders and head, and left the child in the bed. Mr. Euſtace expreſſed great aſtoniſhment at the ſudden change, but I aſſured him that I could claim no other merit on account of this delivery, except that I had not impeded an effect which was wholly produced by the pains.

This child was alſo dead, but the mother recovered in the moſt favourable manner.

In all theſe caſes, the women were at the full period of utero-geſtation, and the children were of the uſual ſize.

Other caſes of the ſame kind have occurred to me, and with the hiſtories of ſeveral, varying in the time or manner in which the evolution of the child was made, I have lately been favoured by gentlemen of eminence in the profeſſion. But theſe these are sufficient to prove the fact, that in cases in which children present with the arm, women will not necessarily die undelivered, though they are not assisted by art.

With respect to the benefit we can, in practice, derive from the knowledge of this fact, I may be permitted to observe, that the custom of turning and delivering by the feet in presentations of the arm, will remain necessary and proper, in all cases, in which the operation can be performed with safety to the mother, or give a chance of preserving the life of the child. But when the child is dead, and when we have no other view but merely to extract the child, to remove the danger thence arising to the mother, it is of great importance to know that the child may be turned spontaneously, by the action of the uterus. If we avail ourselves of that knowledge, the pain and danger which sometimes attend the operation of turning a child, may be avoided. Nor would any person versed in practice, fixing upon a case of preternatural presentation, in which he might expect the child to be turned spontaneously, be involved in difficulty, if from a defect of the pains or any other cause, he should be disappointed in his expectations. Nor would would the ſuffering or chance of danger to the patient be increaſed by ſuch proceeding.

To this account, I beg leave to add, that the caſes of which I have been informed, now amount to near thirty, and that in one of them, which was under the care of our friend Dr. Garthſhore, the child was born alive.

I am, dear Sir,

Your moſt humble ſervant,

THOMAS DENMAN.

Old Burlington-ſtreet

Dec. 7, 1783.

